# Regulation of Liquid Self‐Transport Through Architectural‐Thermal Coupling: Transitioning From Free Surfaces to Open Channels

**DOI:** 10.1002/advs.202412483

**Published:** 2025-01-31

**Authors:** Qingwen Dai, Chengxuan Du, Wei Huang, Xiaolei Wang

**Affiliations:** ^1^ National Key Laboratory of Helicopter Aeromechanics Nanjing University of Aeronautics & Astronautics Nanjing 210016 China; ^2^ College of Mechanical and Electrical Engineering Nanjing University of Aeronautics & Astronautics Nanjing 210016 China

**Keywords:** hierarchical structure, open channel, Self‐transport, thermal gradient

## Abstract

In this work, the regulation of liquid self‐transport is achieved through architectural and thermal coupling, transitioning from free surfaces to open channels. Hierarchical structures inspired by the skin of a Texas horned lizard are designed, with the primary structure of wedged grooves and the secondary structure of capillary crura. This design enables advantages including long‐distance self‐transport, liquid storage and active reflux management on free surfaces, directional transportation, synthesis and detection of reagents in confined spaces, as well as controllable motion and enhanced heat dissipation in open channels. The regulation capacity can be precisely controlled by adjusting the secondary capillary crura and external thermal gradients. The regulation mechanism is further elucidated through microscopic flow observation and a deduced theoretical model. The proposed structures are expected to introduce a new concept for designing lubrication systems, microfluidic chips, methods for chemical synthesis, and heat transfer in the future.

## Graphical abstract

Inspired by the skin of the Texas horned lizard, hierarchical structures composed of primary wedged grooves and secondary capillary crura are proposed, and the regulation of liquid self‐transport is achieved through architectural and thermal coupling, transitioning from free surfaces to open channels.

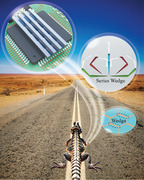

## 1 Introduction

In nature, liquid transport facilitates the transfer of mass and energy between nonliving and living matter.^[^ ^1^, ^2^, ^3^, ^4^, ^5^, ^6^, ^7^, ^8^, ^9^ ^]^ In recent decades, controllable liquid transport has rapidly advanced due to its significant potential in scientific and engineering applications such as microfluidics,^[^ ^10^ ^]^ chemical synthesis,^[^ ^11^ ^]^ energy recovery,^[^ ^12^, ^13^ ^]^ enhanced heat transfer,^[^ ^14^, ^15^ ^]^ and lubrication systems.^[^ ^16^ ^]^ The Marangoni effect,^[^ ^17^ ^]^ where an unbalanced surface tension gradient in a liquid causes motion from low to high‐tension regions, is key to this. Researchers have developed two main approaches for regulating liquid self‐transport: applying external electric,^[^ ^18^, ^19^, ^20^, ^21^, ^22^ ^]^ thermal,^[^ ^23^, ^24^, ^25^ ^]^ magnetic,^[^ ^26^, ^27^ ^]^ or optical^[^ ^28^, ^29^, ^30^ ^]^ fields to create a surface tension gradient, and constructing micro‐nano structures to form a wettability gradient on solid surfaces.^[^ ^31^, ^32^, ^33^, ^34^, ^35^, ^36^, ^37^ ^]^ The latter is often preferred as it doesn't require additional devices.

Nature offers numerous inspirations for designing micro‐nano structures. Through the processes of natural selection and evolution, many organisms have developed specialized surfaces that exhibit remarkable liquid transport capabilities to adapt to environmental changes.^[^ ^38^, ^39^, ^40^ ^]^ For instance, the conical tip of a cactus captures droplets from mist and transports them from narrow to wide end.^[^ ^41^ ^]^ The Janus structure on the tip of pine needles can merge small droplets and direct them to the needle's root.^[^ ^42^ ^]^ The skin of the Texas horned lizard, a honeycomb‐shaped wedge array, can provide directional, passive liquid transport as a model for a biomimetic liquid diode^[^ ^43^ ^]^

The driving force behind the self‐transport capabilities of these biological surfaces is the asymmetric Laplace pressure. The wedged or patterned structures create a curvature difference across opposing sides of a liquid, resulting in an unbalanced Laplace pressure that causes spontaneous flow.^[^ ^44^, ^45^, ^46^ ^]^ Inspired by these natural systems, Alheshibri et al.^[^ ^47^ ^]^ fabricated a wedge‐shaped hydrophilic region against a hydrophobic background on a solid surface and experimentally observed the liquid self‐transport process. Dai et al.^[^ ^48^ ^]^ designed superoleophobic surfaces with wedge‐shaped superoleophilic grooves to control the thermocapillary migration. Additionally, researchers have developed theoretical models using Lattice‐Boltzmann^[^ ^49^ ^]^ and molecular dynamics^[^ ^50^ ^]^ to elucidate the transport mechanism.

The wettability difference and the divergent angle of a wedged structure are key parameters influencing its self‐transport capability.^[^ ^51^ ^]^ In such structures, the wedged area is typically hydrophilic, while the surrounding area is hydrophobic. However, there is a limitation to the wettability difference since the maximum difference in contact angles is less than 180°. Although increasing the divergent angle can improve self‐transport efficiency, it also enlarges the hydrophilic area, leading to significant liquid loss and a reduction in the overall transport effectiveness. These limitations of a single wedge groove become apparent when dealing with liquid transport on complex surfaces, confined spaces, or in open channels. Song et al.^[^ ^52^ ^]^ demonstrated long‐distance transport of underwater bubbles using a continuous gradient surface by connecting multiple wedged structures. Hou et al.^[^ ^53^ ^]^ created patterned superhydrophilic wedged grooves on a superhydrophobic surface, enabling water condensation collection against gravity. Liu et al.^[^ ^54^ ^]^ optimized the design of connected wedged structures and developed a series of cycloidal wedge grooves to enhance water transport.

Patterned wedge‐shaped structures indeed offer significant advantages for driving liquid flow. However, most research has focused on enhancing the transport of water droplets, with less attention given to self‐transport regulation for other liquids like mineral or lubricating oils, which differ in viscosity and surface tension. Accurately controlling the flow of these varied liquids remains a major challenge. Currently, the directional transport of liquids in open channels is a burgeoning area in microfluidics.^[^ ^55^, ^56^, ^57^, ^58^ ^]^ The flow state of the liquid in the open flow channel is different from that on the free surface. There are two more side walls in the open channel, and the liquid flow is related to the spontaneous capillary flow and the depth‐to‐width ratio of the section.^[^ ^59^, ^60^, ^61^, ^62^ ^]^ It remains uncertain whether patterned structures can effectively regulate liquid flow in open channels. Successfully addressing this could enable long‐distance transportation of large volumes of liquid within such channels.

In this study, hierarchical structures inspired by the skin of a Texas horned lizard were proposed to regulate liquid self‐transport coupling thermal effect. The hierarchical structures can effectively drive the movement of typical lubricants. By designing an appropriate chamfer radius at the connection points of the wedged units, we were able to significantly enhance self‐transport performance. The regulation capacity could be precisely controlled by adjusting the secondary capillary crura and thermal gradient. Experimental confirmation showed their potential applications in lubrication under starved conditions, reagent synthesis and detection, long‐distance transportation, and enhanced heat dissipation in open channels. The regulation mechanism was further elucidated through microscopic flow observations. The proposed hierarchical structures are expected to introduce a new concept for designing lubrication systems, microfluidic chips, chemical synthesis, and enhanced heat transfer methods in the future.

## 2 Results and Discussion

### 2.1 Long‐Distance Self‐Transport Capability of Designed Surface

**Figure 1:**
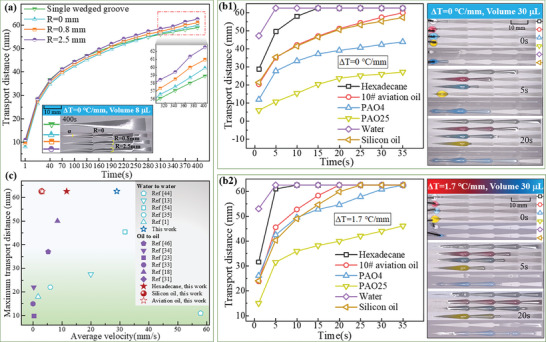
a) Relationship between distance and time for on surfaces with wedged groove patterns with increasing chamfer radii (R). Performance of six different lubricants on surfaces with a wedged groove pattern (R = 2.5 mm), exhibiting a thermal gradient of (b1) ΔT = 0 °C mm^−1^, and (b2) ΔT = 1.7 °C mm^−1^. c) Comparison of the maximum transport distance and average velocity between this work and opening literature for water‐to‐water and oil‐to‐oil systems.

Three types of wedged groove patterns were fabricated, each with increasing chamfer radii (R) of 0, 0.8, and 2.5 mm at the connecting region (Design details on the parameters were provided in Figure S1a, Supporting Information). For comparison, a single wedged groove was also tested. The length (L) of the designed structure was 62.58 mm, with a divergent angle (α) of 7°. The wedged groove area was oleophilic, while the surrounding area was oleophobic, with contact angles of ≈1° and 158°, respectively. **Figure** **1**a illustrates the self‐transport performance using 8 µL paraffin oil droplets. The patterned wedged grooves exhibited superior self‐transport capacity compared to the single one. Chamfer radius is a positive correlation with the transport performance, thus, a chamfer radius of 2.5 mm was selected in the subsequent sections. Note that the self‐transport capacity is related to the wettability and wedged angle,^[^ ^51^, ^52^, ^53^, ^63^ ^]^ increasing the size of the openings at both ends of the wedge shape would not affect the self‐transport capacity, but it can transport more volume of liquid. The architectural‐thermal coupling of liquid self‐transport capacity was further validated by different lubricants. As shown in Figure 1b1, the designed surface demonstrated controllable self‐transport for Hexadecane, 10# aviation hydraulic oil, Poly‐alfa‐olefins 4 (PAO4), PAO25, water, and silicon oil. PAO25 exhibited a shorter transport distance due to its higher viscosity (Movie S1, Supporting Information). When a thermal gradient (ΔT = 1.7 °C mm^−1^) was applied (Figure 1b2), the self‐transport capacity improved significantly for all tested liquids. For instance, the transport distance of PAO25 increased from 27.17 to 46.18 mm within 35 s (Movie S2, Supporting Information).

Figure 1c presents a comparison of the maximum transport distance and average velocity between this work and the opening literature for water‐to‐water and oil‐to‐oil systems, specifically regarding the wedged‐like structures. It is confirmed that the proposed structures can realize a long‐distance self‐transport for different lubricants.

### 2.2 Capacity for Liquid Storage and Active Reflux Management

**Figure 2:**
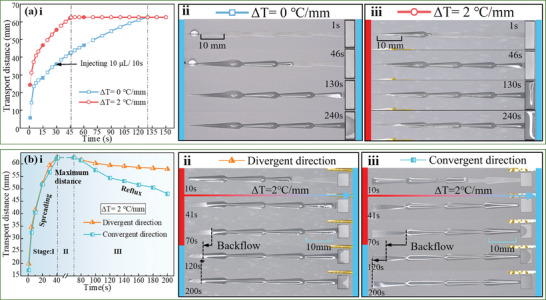
Liquid storage performance and active reflux management of the designed surfaces: a) The transport distance and detailed transportation process for liquid storage under a thermal gradient of ΔT = 0 °C mm^−1^, and ΔT = 1.7 °C mm^−1^. b) Active reflux management performance under thermal gradients of ±2 °C mm^−1^.

The performance of liquid storage and active reflux on the designed surfaces was evaluated. As illustrated in **Figure** **2**a, 10 µL droplets of paraffin oil were applied to the surface every 15 s, totaling 50 µL. Without a thermal gradient, the majority of the liquid remained in the wedged grooves and did not fully flow into the pool after 240 s. However, when a thermal gradient of ΔT = 2 °C mm^−1^ was introduced in the divergent direction of the wedged groove, the liquid flowed rapidly and reached the pool area in just 46 s, significantly faster than under simple architectural conditions. Figure 2b shows the active reflux performance of the liquid on the designed surfaces (the reflux distance equals 62.58 mm minus the movement distance of the black arrow, as shown in Figure 2b(ii,iii). Two thermal gradients were applied: 2 °C mm^−1^ in the divergent direction and −2 °C mm^−1^ in the convergent direction. The process can be divided into three stages. Stage I: For ΔT = ±2 °C mm^−1^, the liquid reached the pools on the cold side within 41 s. Stage II: As the transport continued, liquid flowed from the wedged grooves into the pools between 41 and 70 s. Stage III: After the thermal gradients were removed at the 70s, the liquid exhibited different behaviors based on the direction of the gradient. For ΔT = 2 °C mm^−1^ (divergent direction), the liquid flowed back slightly. In contrast, for ΔT = −2 °C mm^−1^ (convergent direction), the main portion of the liquid quickly flowed back to its initial position from right to left.

Based on the experiments conducted, it is confirmed that architectural‐thermal coupling offers a controllable self‐transport capacity. The designed surface effectively returns lubricants to the initial region, even when faced with a thermal gradient. This capability could be utilized to transport or collect lubricants to the designated area within advanced lubrication systems that encounter varying thermal gradients.

### 2.3 Directional Self‐Transport Capability in Confined Spaces

**Figure 3:**
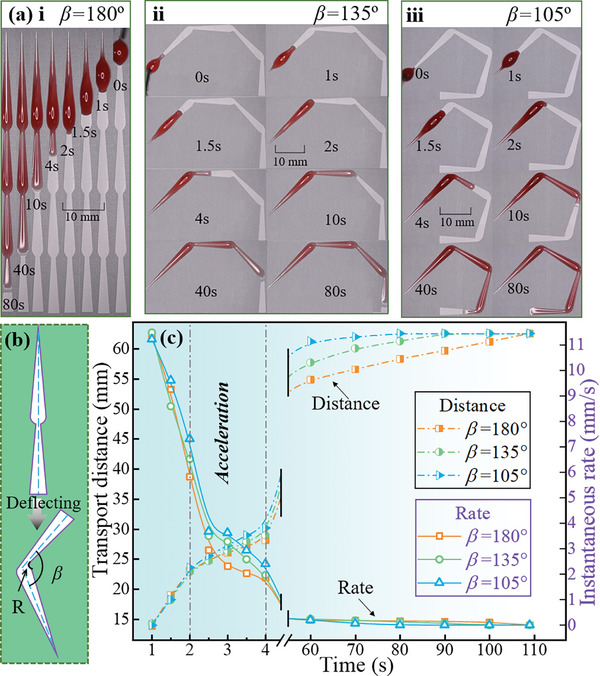
Directional self‐transport capability in confined spaces: a) Detailed transportation process on wedged groove patterns with varying deflection angles (*β*) of 180°, 135°, and 105°. b) Design schematic of a chamfered series of wedged grooves featuring a deflection angle. c) Results showing transport distance and instantaneous rate.

Compared to straight wedged groove patterns, incorporating a deflection angle (*β*) between each wedged groove allows for directional self‐transport capacity in confined spaces. As illustrated in **Figure** **3**a,b, wedged groove patterns with varying deflection angles (*β*) of 180°, 135°, and 105° were fabricated. The geometric dimensions of each unit (wedged groove) remained unchanged, with the only modification being the introduction of an inner chamfer radius (R = 0.5 mm) at the junction region to facilitate the bent pattern (more details were provided in Figure S1b, Supporting Information). The results of the directional self‐transport are shown in Figure 3c. Velocity is a vector, since its direction is changing due to varying deflection angles, here, instantaneous rate (just magnitude) is used for comparison. The transport distance is inversely correlated with the deflection angle (*β*). Specifically, when comparing *β* = 180° to *β* = 105°, the time required to reach the maximum distance is reduced by 36 s. Additionally, the instantaneous rate indicates that there is no significant difference among all designed surfaces before reaching the first junction region.

It is interesting to see that once the liquid flowed over the deflection angle at ≈2 s, a significant difference was observed, revealing an interesting phenomenon: the liquid exhibited an acceleration process around the junction region. To understand the acceleration mechanism, the microscopic transport process of the liquid flowing through the connection region was analyzed.

**Figure 4:**
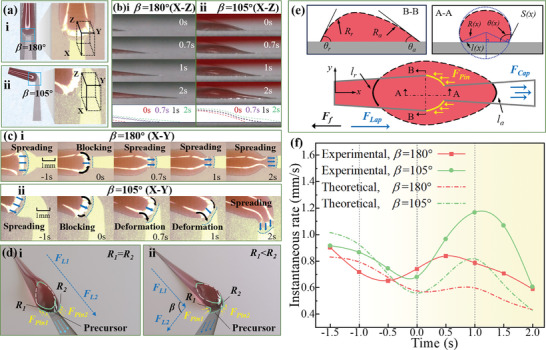
Acceleration mechanism at the junction region: Microscopic self‐transport processes of lubricant as it flows through the junction region illustrated in a) a coordinate diagram, b) a side view, and c) a bird's eye view. d) The forces present at the junction region and e) a simplified theoretical model. f) The experimental results and theoretical predictions of the instantaneous rate.

**Figure** **4**a illustrates the microscopic self‐transport process at the junction region. The second connection point was designated as the reference, and the moment when the liquid precursor reached the tangent of the chamfer radius was defined as 0 s. A 3D coordinate system was established to provide a perspective on the different sections of paraffin oil flowing through the junction region. Figure 4b presents a side view (X‐Z coordinate) of the detailed self‐transport process. When *β* equals 180°, the convex meniscus becomes increasingly flatter. In contrast, at *β* equal to 105°, the precursor rapidly elongates, and the level height gradually increases after passing through the junction region. The extracted contour curve of the liquid demonstrates the differences in the surface profile of the convex meniscus. From a bird's eye view (X‐Y coordinate) in Figure 4c, it is evident that the liquid precursors on these two surfaces exhibited convex menisci and were obstructed at the junction area at 0 s. For *β* = 180°, the continuous accumulation of liquid in the junction region hindered the transport process, resulting in only a thin film flowing through. In contrast, for *β* = 105°, the transport behavior near the inner chamfer was more prominent than that near the outer chamfer. This led to the elongation of the precursor, preventing liquid accumulation at the junction region. Consequently, the convex meniscus became more pronounced during the turning process, initiating an acceleration phase.

Based on the microscopic process described above, we can gain insight into the acceleration mechanism. As illustrated in Figure 4d‐i, the Laplace pressure (resulting from the convex meniscus) serves as the driving force for self‐transportation. For *β* = 180°, the inner and outer chamfer radii at the junction region are equal (*R_1_* = *R_2_*), causing the liquid to encounter equivalent pinning resistance (*F_pin1_* = *F_pin2_*) as it flows over the connection point. In contrast, for *β* = 105° (Figure 4d‐ii), the inner and outer chamfer radii at the structural connection are unequal (*R_1_* <*R_2_*), leading to a disparity in pinning resistance. Meanwhile, the contact line of the liquid precursor becomes elongated and bent, generating additional Laplace pressure in that region.

This asymmetry in pinning resistance, combined with the extra Laplace pressure, accelerates the self‐transport process. The external force (*F*) acting on the liquid can be expressed as follows:

(1) $$F=F_{Lap}+F_{Cap}-F_{Pin}-F_{f}$$

where *F_Lap_* represents the Laplace driving force (mainly attributed by the micro wedged groove, that is, the curvatures at the right and left sides), *F_Cap_* signifies the structure‐induced capillary driving force (mainly attributed by the micro‐nano roughness), *F_Pin_* indicates the pinning resistance at the triple‐phase contact line, and *F_T_* denotes the frictional resistance.

Referring to the inserts in Figure 4e, the Laplace pressure (Δ*P*) can be determined via the Young‐Laplace equation:

(2) $$\Delta P=\gamma_{SL}\;\left(\frac{1}{R_{a}}+\frac{1}{R_{r}}\right)$$

where *γ_SL_* is the surface tension of a liquid (*γ_SL_* can be simplified to *γ*), *R_a_* and *R_r_* represent the radii of curvature of the liquid. As shown in Figure 4e, the relationship between *R*(*x*) and θ(*x*) can be written as follows:

(3) $$\left\{\begin{array}{c} \;\cos\left(\theta -\frac{\pi }{2}\right)=\frac{\frac{l\left(x\right)}{2}}{R\left(x\right)}\;\\ R\;\left(x\right)=\frac{l\left(x\right)}{2\sin\theta \left(x\right)}\; \end{array}\right.$$

where *l*(*x*) is the length of the contact line.

Substituting Equation 3 into Equation 2, the Laplace pressure (Δ*P*) can be written as:

(4) $$F_{Lap}=\Delta PS\;\left(x\right)=2\gamma \left(\frac{\sin\theta_{a}}{l_{a}}+\frac{\sin\theta_{r}}{l_{r}}\right)S\left(x\right)$$

where *S(x)* represents the cross‐sectional area of the liquid in the direction of the *y*‐axis.

When considering the solid‐liquid capillary forces involved in the self‐transport process, the capillary driving force *F_Cap_* along the depth of the wedged groove can be determined as:^[^ ^64^ ^]^

(5) $$F_{Cap}=\gamma l_{a}\cos\frac{\alpha }{2}h$$

where *α* represents the divergent angle, and *h* denotes the depth of the wedged structure.

The pinning resistance at the triple‐phase contact line, denoted as *F_Pin_*, can be calculated using the Furmidge equation.^[^ ^65^ ^]^:

(6) $$F_{Pin}=\gamma l_{a}\left(\cos\theta_{a}-\cos\theta_{r}\right)$$

where θ_*a*_ and θ_*r*_ represents the advancing and receding contact angle, respectively. The frictional resistance (*F_f_*) can be written as:

(7) $$F_{f}=\frac{3\mu L}{l_{a}h^{3}}\;\cos\frac{\alpha }{2}$$

where μ represents the viscosity, *L* represents the length of the hydrophilic region.

Substituting Equations (4), (5), (6), (7) into Equation 1, the external force (*F*) acting on the liquid can be obtained:

(8) $$\begin{array}{ccc} & & F=2\gamma \frac{l_{r}\sin\theta_{a}+l_{a}\sin\theta_{r}}{l_{a}l_{r}}S\left(x\right)+\gamma l_{a}\left(\cos\frac{\alpha }{2}h-\cos\theta_{a}+\cos\theta_{r}\right)\\ & & -\frac{3\mu L}{l_{a}h^{3}}\cos\frac{\alpha }{2} \end{array}$$

Given that the flow rate is low (indicating a small Reynolds number) and the liquid has low viscosity, the self‐transport process can be considered laminar flow. Therefore, the inertia term in the Navier‐Stokes equation can be neglected. Focusing on the directional movement along the *x*‐axis, the simplified 2D Navier‐Stokes equation can be expressed as follows:

(9) $$\frac{\partial^{2}u}{\partial y^{2}}=\frac{1}{\mu }\;\frac{\partial P}{\partial x}$$

Combing Equations (8) and (9), the theoretical instantaneous rate (*u*) can be obtained by integrating Equation 9 twice with the boundary conditions (*u* = 0 at *x_1_* = 0, *y_1_* = 0 and *x_2_* = 62.58 mm, *y_2_* = 2.5 mm):

(10) $$u=\frac{1}{2\mu }\;\frac{y^{2}}{x}\frac{F}{S\left(x\right)}+C_{exp}y$$

where *C_exp_* is the coefficient related to the initial volume of liquid, *y* represents the length of the contact line. By substituting all variables into Equation 10, the instantaneous rate (*u*) can be calculated. As illustrated in Figure 4f, although the amplitude of the experimental results is slightly higher than that of the theoretical predictions, the general trends of the experimental and theoretical instantaneous rates (*u*) are remarkably similar. By defining the time at which the liquid reaches the second junction region as 0 s, the bent structure induces an acceleration effect that is observable within the time interval of 0–2 s.

### 2.4 Synthesis and Detection of Reagents in a Confined Space

**Figure 5:**
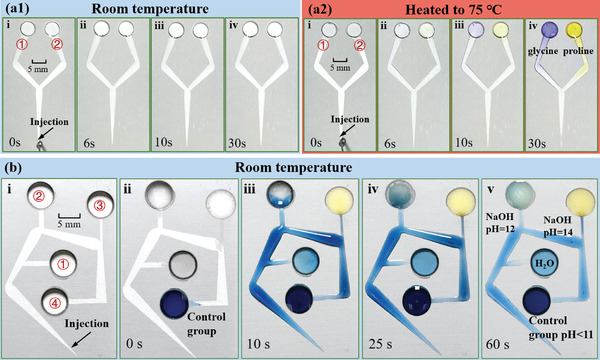
Synthesis and detection of reagents in a confined space: amino acid chromogenic reaction at room temperature a1) and heated to 75 °C a2) on surfaces with “double halberd” structures. b‐i) Design diagram of the bent structure with regions ①‐④ marked for detection. b‐ii–v) Flow charts depicting the transportation of the blue indigo disulfonate sodium‐glucose solution to the four designated detection areas.

**Figure** **5**a illustrates the potential application for amino acid chromogenic reaction in a limited space with the designed “double halberd” structures. 80 µL ninhydrin aqueous solution (4 wt.%, glacial acetic acid: water = 100:3) was used as the detection solution (Movie S3, Supporting Information). The areas marked ① and ② were randomly dropped 30 µL liquids of proline (1 wt.%) and glycine (1 wt.%) aqueous solutions. As shown in Figure 5a1, the detection solution was rapidly transported to the detection areas within 6 s, but there was no color change until 30 s. When the surface was heated to 75 °C (Figure 5a2), obvious color changes appeared at the detection areas at 10 s, and the colors were stable after 30 s. It can be confirmed that purple area ① was glycine solution and yellow area ② was proline solution, respectively.

Figure 5b illustrates another demonstrative experiment in a limited space for reagent synthesis and detection. Notably, the sodium indigo disulfonate‐glucose aqueous solution will exhibit different colors when mixed with alkaline liquids of varying pH levels, serving as a demonstrative experiment for acid‐base identification. In Figure 5b‐i, the diagram of the designed structure was presented, with the areas marked ①, ②, ③, and ④ representing four different alkaline liquids to be tested. Initially, ultrapure water (colorless), a NaOH aqueous solution (colorless, pH = 12.4), and another NaOH aqueous solution (colorless, pH = 14) were randomly added to three of the areas (①, ②, and ③) designated for detection. As a control, the sodium indigo disulfonate‐glucose aqueous solution (blue, at pH < 11) was added to area ④, as shown in Figure 5b‐ii. Subsequently, 60 µL of the blue sodium indigo disulfonate‐glucose aqueous solution was applied at the entrance. After ≈10 s, the detection solution self‐transported to the four designated areas, as illustrated in Figure 5b‐iii. Following the reaction process, the results were as follows: Region ① displayed a light blue color, indicating ultrapure water (pH < 11); Region ② turned light green, representing a NaOH aqueous solution with a pH of 12.4 (11.4 < pH < 13); and Region ③ became yellow, corresponding to a NaOH aqueous solution with a pH of 14 (pH > 13). These results are shown in Figure 5b(iv,v).

This indicates that the proposed structures can effectively facilitate reagent synthesis and detection within a limited space, especially when the reaction requires to be heated. These break through the limitations of limited space and the inability to heat the reaction.

### 2.5 Controllable Self‐Transport Capacity of Hierarchical Structures Patterns

**Figure 6:**
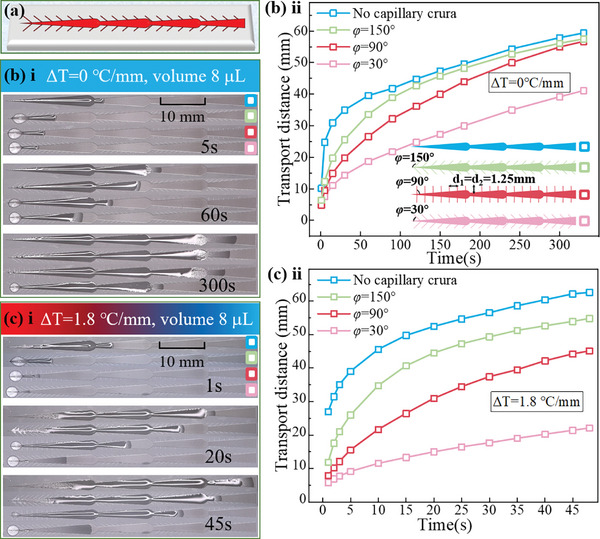
Controllable self‐transport capacity of hierarchical structure patterns: a) Design schematic of wedged grooves featuring capillary crura. Detailed self‐transport distance and transportation process on surfaces with capillary crura with orientation angles (*φ*) of 150°, 90°, and 30°, under two different thermal gradients: b) ΔT = 0 °C mm^−1^, and c) ΔT = 1.8 °C mm^−1^.

The structures mentioned above demonstrated excellent self‐transport capacity, this capacity could not be precisely controlled as needed. To achieve this goal, wedged grooves were regarded as the primary structure, and secondary structures in the form of capillary crura were fabricated around the wedged grooves. As illustrated in **Figure** **6**a,b, capillary crura patterns with different orientation angles (*φ*) of 150°, 90°, and 30° were prepared, while keeping all other parameters constant. The self‐transport capacity of 8 µL silicone oil on the hierarchical structures was evaluated with and without external thermal gradients. Single wedged grooves exhibited the fastest self‐transport distance when ΔT = 0 °C mm^−1^ (Figure 6b). The inclusion of capillary crura patterns allowed for controllable self‐transport performance, with controllability ranking in descending order for *φ* = 150°, 90°, and 30°. When subjected to a thermal gradient (ΔT = 1.8 °C mm^−1^), all corresponding self‐transport distances increased; however, the overall trends and controllability of the designed hierarchical structures remained unchanged, as shown in Figure 6c (Movie S4, Supporting Information).

The experimental results demonstrated that designing capillary crura provides an effective method for controllable self‐transport capability. The overall self‐transport distances ranked in descending order as follows: structures without capillary crura, followed by those with capillary crura at *φ* = 150°, 90°, and 30°.

### 2.6 Long‐Distance Directional Self‐Transport in Open Channels

**Figure 7:**
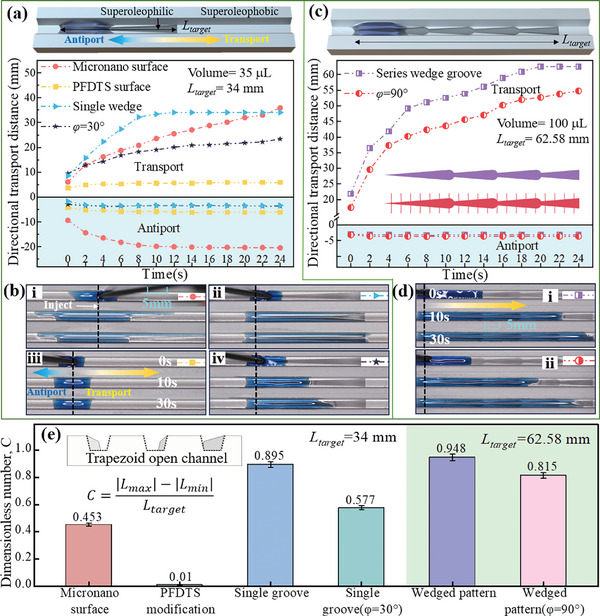
The transport distance and processes observed on various surfaces: a,b) Four distinct surfaces are analyzed, including micronano structures, PFDTS modification, single wedged groove, and single wedged groove featuring capillary crura (*φ* = 30°). c,d) Two surfaces are examined, highlighting the wedged groove pattern and wedged groove pattern with capillary crura (*φ* = 90°).,e) Self‐transport capacity in open channels evaluated via a dimensionless number (*C*).

The hierarchical structure patterns can be further utilized for directional self‐transport in open channels. Since silicone oil has the lowest surface tension, it was chosen in the following experiments to better reflect the influence of the designed structures. As illustrated in **Figure** **7**a,b, single wedged groove, and single wedged groove decorated with capillary crura (*φ* = 30°) were prepared, along with micronano structures (after a boiling process) and PFDTS modification (after low surface energy treatment) as control groups. The maximum transport distance (*L_target_*) achieved was 34 mm, utilizing 35 µL of silicone oil. In the open channel with a single wedged groove, silicone oil was able to transport directionally and rapidly forward to 34 mm within 12 s (blue line, Figure 7b‐ii). Decorating the groove with capillary crura (φ = 30°) resulted in a reduced flow rate (black line, Figure 7b‐iv). The silicone oil spread in both directions within the channel containing micronano structures (red line, Figure 7b‐i), while it remained stationary in the PFDTS‐modified channel (yellow line, Figure 7b‐iii) (Movie S5, Supporting Information).

Note that single wedged grooves have structural limitations; decreasing the divergent angle can extend their length but at the expense of weakening their self‐transport capacity. Therefore, hierarchical structure patterns were employed for long‐distance transportation in open channels. As shown in Figure 7c,d (Movie S6, Supporting Information), both wedged groove patterns and wedged groove patterns with capillary crura (*φ* = 90°) were prepared, achieving a maximum transport distance (*L_target_*) of 62.58 mm. The results confirm that the silicone oil can reach the target transport distance of 62.58 mm within 24 s (purple line, Figure 7d‐i), and the addition of capillary crura (*φ* = 90°) provides the capability for flow rate control (red line, Figure 7d‐ii). The long‐distance directional self‐transport capacity in open channels is evaluated by involving a dimensionless number, *C*, which is defined as $C=\frac{|L_{max}\left|-\right|L_{min}|}{L_{target}}$ (For a specific liquid, *L_max_* and *L_min_* refer to the maximum and minimum values in the *y*‐axis in Figure 7a,c). As illustrated in Figure 7e, the wedged groove pattern demonstrates the best long‐distance directional self‐transport capacity and controllable flow rates can be achieved by incorporating capillary crura.

### 2.7 Enhanced Heat Dissipation in Open Channels

**Figure 8:**
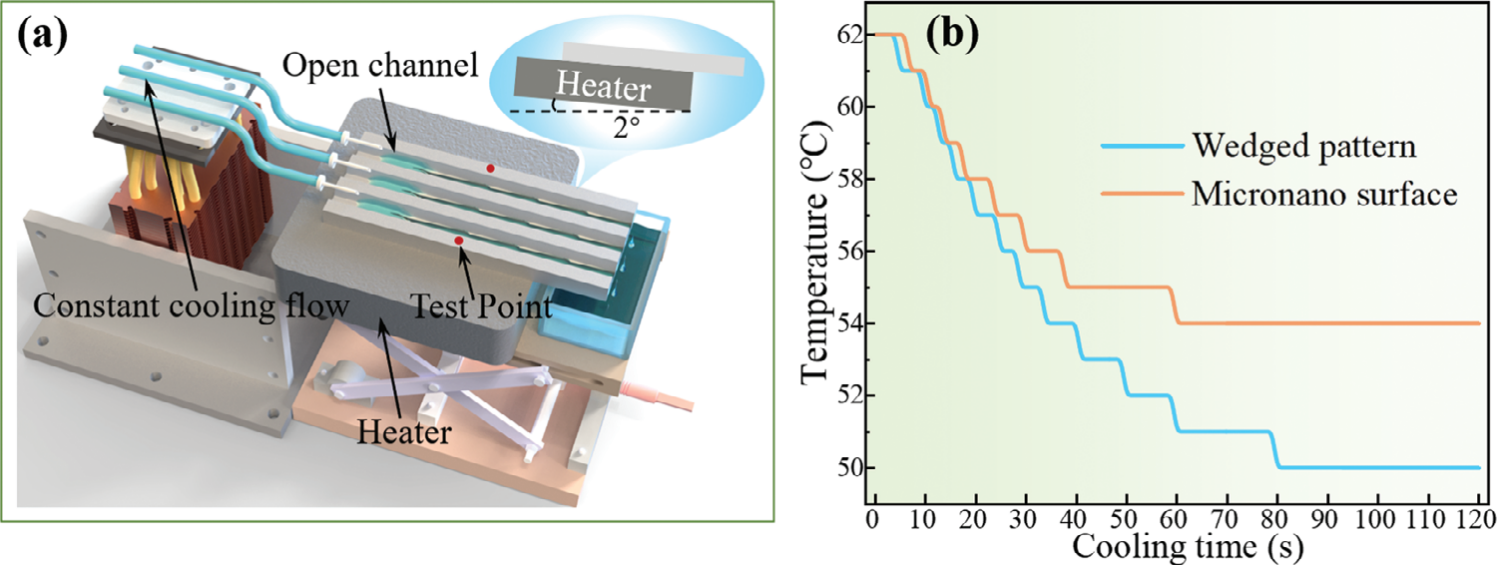
a) Diagram of enhanced heat dissipation experimental device. b) Temperature‐cooling time relationship in open channels with micronano structure and wedged pattern.

The open channel with an optimized surface pattern can enhance the heat dissipation effect of the liquid in the channel. As shown in **Figure** **8**, a constant power heating plate is used to heat the bottom 3/4 of the open channel, maintaining its temperature at 62 °C. The aluminum plate is tilted at 2° to increase the flow rate of the liquid, and a J‐type thermocouple is used to measure the temperature on its surface. A micro constant flow pump and three syringes are used to inject cooling water at a constant rate of 15° into each channel, with the water flow rate set to 28.75 µL s^−1^, continuous injection for 120 s. The detailed data from the heat dissipation experiment is shown in Figure 8b. The open channel with a micronano rough surface reduced the temperature of the aluminum plate from 62 to 54 °C in the 60s, after that the aluminum plate temperature remained constant, while the stable surface temperature of the open channel with the optimized pattern was just 50 °C.

The results indicate that the optimized pattern accelerated the flow of cooling water, allowing the liquid that had exchanged heat to promptly carry away the heat from the channel surface. It is believed that structure design in open channels can enhance the heat dissipation effect, which has potential applications in finned heat exchangers, electronic components, and liquid cooling fields. Note that fabricating the proposed structure on a large area surface with connected open channels will no longer need tilting the system.

## 3 Conclusion

This study introduces an innovative concept for liquid self‐transport, leveraging architectural‐thermal coupling to transition from free surfaces to open channels. We designed and optimized hierarchical structures composed of primary wedged grooves and secondary capillary crura. These hierarchical structures effectively facilitate the movement of lubricants with varying viscosities and surface tensions, enabling several key functions. Long‐distance self‐transport: the design allows for efficient transport of liquids over considerable distances, addressing challenges where traditional methods may fall short. Liquid storage and active reflux management: the structures enable effective liquid storage and management of reflux on free surfaces, enhancing overall system efficiency. Directional transportation: the design supports the directional flow of liquids, which is crucial for applications such as microfluidics and chemical synthesis. Reagent synthesis and detection: the capability to control liquid flow in confined spaces provides opportunities for reagent synthesis and precise detection. Controllable motion in open channels: with the secondary structures in the form of capillary crura, we can regulate liquid movement within open channels, allowing the system to adapt to various operational conditions. Enhanced heat dissipation: compared to a normal channel, the optimized surface pattern in the open channel can enhance cooling capacity. The regulatory mechanism governing these functions was further elucidated through microscopic flow observations and theoretical modeling, which deepened our understanding of the underlying principles. Experimental validation underscores the practicality of these hierarchical structures for applications in lubrication under starved conditions, chemical reagent synthesis, efficient transportation, and enhanced heat transfer within open channel systems.

## 4 Experimental Section

### Materials

All substrates were constructed from pure aluminum plates with dimensions of 75 × 30 × 5 mm. For surface modification, 1H, 1H, 2H, 2H‐perfluorodecyltrichlorosilane (PFDTS) was employed. Various lubricants including n‐hexadecane (viscosity, contact angle, surface tension: 1.5 cSt, 159°, 34.9 mN m^−1^), 10 aviation hydraulic oil (10 cSt, 153.5°, 32 mN m^−1^), PAO4 (19 cSt, 153°, 29 mN m^−1^), PAO25 (238 cSt, 154°, 28 mN m^−1^), paraffin oil (13.4 cSt, 158°, 32 mN m^−1^), silicone oil (10 cSt, 145°, 22 mN m^−1^), and water (1 cSt, 165°, 72 mN m^−1^), were used. All chemical reagents were analytically pure and purchased from Aladdin, China, and were used as received in this study.

### Fabrication

**Figure 9:**
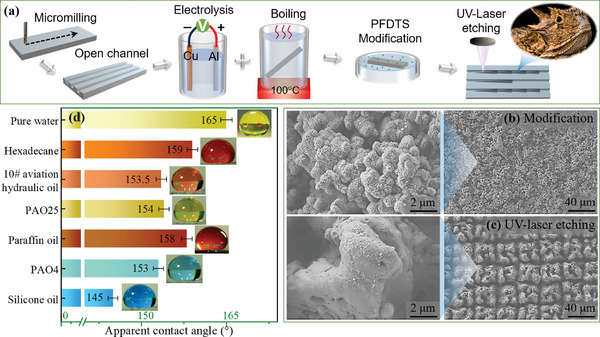
a) Fabrication process of hierarchical structures on Al surfaces. b,c) SEM images of the prepared surfaces after PFDTS modification and UV‐laser etching process. d) The apparent contact angle of seven typical lubricant oils on oleophobic surfaces.

**Figure** **9**a illustrates the preparation process of hierarchical structures on Al surfaces. The aluminum surface was polished using sandpapers with mesh sizes P480, W50, and W20 to remove the oxide layer. For the open channels, an inverted trapezoidal open channel was milled into the surfaces using a micro‐milling machine equipped with a specially designed trapezoidal tool (detailed dimensions are provided in Figure S2, Supporting Information). The cross‐section of the flow channel was an inverted isosceles trapezoid, which ensures that the laser does not damage the side walls of the flow channel when a suitable processing pattern was designed. Following this, the surfaces were ultrasonically cleaned in ethanol for 5 min and then etched through anodic electro‐dissolution (0.1 mol L^−1^ sodium chloride, 0.7 A cm^−^^2^ for 7 min). The aluminum was then dipped in boiling water for 40 min for alkaline oxidation, which created hierarchical micro‐ and nanostructures. Subsequently, the surfaces were immersed in a 1 wt.% ethanol solution of PFDTS for 180 min to reduce the surface energy and were dried at 100 °C for 40 min to achieve superoleophobic properties. Superoleophilic regions were created on the superoleophobic surfaces using a UV laser (KY‐M‐UV3L, Wuhan Keyi, China) with the following parameters: power of 3.5 W, frequency of 15 kHz, and speed of 1500 mm s^−1^. This one‐time laser processing achieved an average depth of 11 µm (Contour‐GT, Bruker, USA) (3D morphology of the prepared surface was provided in Figure S3, Supporting Information).

### Characterization

Figure 9b,c present SEM images of the prepared surfaces after PFDTS modification and the UV‐Laser etching process. The smooth aluminum surface was initially hydrophilic. After undergoing electrolysis and boiling, the aluminum surface forms irregular micro‐nano structures. The subsequent application of PFDTS modifies the surface, transforming it into a Cassie‐Baxter wetting state, which results in a super‐oleophobic surface. Laser treatment further roughens the surface, converting it into a super‐oleophilic Wenzel wetting state (The preliminary experimental results are provided in Figure S4, Supporting Information). The on‐demand directional transportation of lubricating oil on the surface or within the open channel can be achieved by leveraging the super‐wetting characteristics of the super‐hydrophobic/super‐oleophilic surface in conjunction with the optimized series gradient pattern. The apparent contact angles of various droplets on the prepared surface were measured using the sessile drop method with a contact angle measuring instrument (SL‐200B, Solon, China), as shown in Figure 9d. A 10 µL droplet was placed on the PFDTS‐modified surfaces, and images were captured after the droplet reached a stable state. The apparent contact angles of n‐hexadecane, No. 10 aviation hydraulic oil, PAO4, PAO25, paraffin oil, silicone oil, and pure water on the low surface energy modified surfaces were recorded as 159°, 153.5°, 153°, 154°, 158°, 145°, and 165°, respectively. In contrast, on the laser‐etched surface, the contact angles of these liquids ranged from 0° to 2°. This indicates that the liquids on this surface achieve two states: super‐hydrophilicity and hydrophobicity. Note that the set temperature was much lower than the boiling point of the used liquids in this study, except for pure water, the liquid evaporation effect was ignored in this study.

In the preliminary tests, various lubricating oils were utilized to assess the flow adaptability and self‐driving capabilities of the optimized chamfered series wedge groove structure. Considering the initial wetting effect, 1 s after droplets contacting the surfaces was defined as the starting time for evaluating the self‐transport performances. To facilitate comparison, this study primarily focused on paraffin oil and silicone oil to investigate the directional transport characteristics of the optimized structural surface and the open flow channel, respectively. The directional transport experiments for all liquids were conducted using a custom‐designed experimental setup (More details are provided in Figure S5, Supporting Information). The macroscopic flow processes were recorded with a digital camera (D750, NIKON, Japan), while the microscopic flow processes were captured using an ultra‐depth‐of‐field microscope (VHX‐600E, Keyence, Japan) and a macro lens (Ultra Macro 2.5‐5.0 × F2.8‐16, LAOWA, China).

## Conflict of Interest

The authors declare no conflict of interest.

## Supporting information



Supporting Information

Supplemental Movie 1

Supplemental Movie 2

Supplemental Movie 3

Supplemental Movie 4

Supplemental Movie 5

Supplemental Movie 6

## Data Availability

The data that support the findings of this study are available from the corresponding author upon reasonable request.
