# Supplementary material for: Regulation of Liquid Self‐Transport Through Architectural‐Thermal Coupling: Transitioning From Free Surfaces to Open Channels
Source: Adv Sci (Weinh). 2025 Jan 31;12(15):2412483. doi: 10.1002/advs.202412483 (PMC12005752; doi:10.1002/advs.202412483)
Supplement: Supplementary file 1 — Supporting Information [file ADVS-12-2412483-s006.docx]

**Supplementary Materials**

**Regulation of Liquid Self-Transport through Architectural-Thermal Coupling: Transitioning from Free Surfaces to Open Channels**

Qingwen Dai^1^^[[1]](#footnote-1)^*, Chengxuan Du^1^, Wei Huang, Xiaolei Wang

College of Mechanical and Electrical Engineering, Nanjing University of Aeronautics & Astronautics, Nanjing 210016, China

**1. Figure Captions**


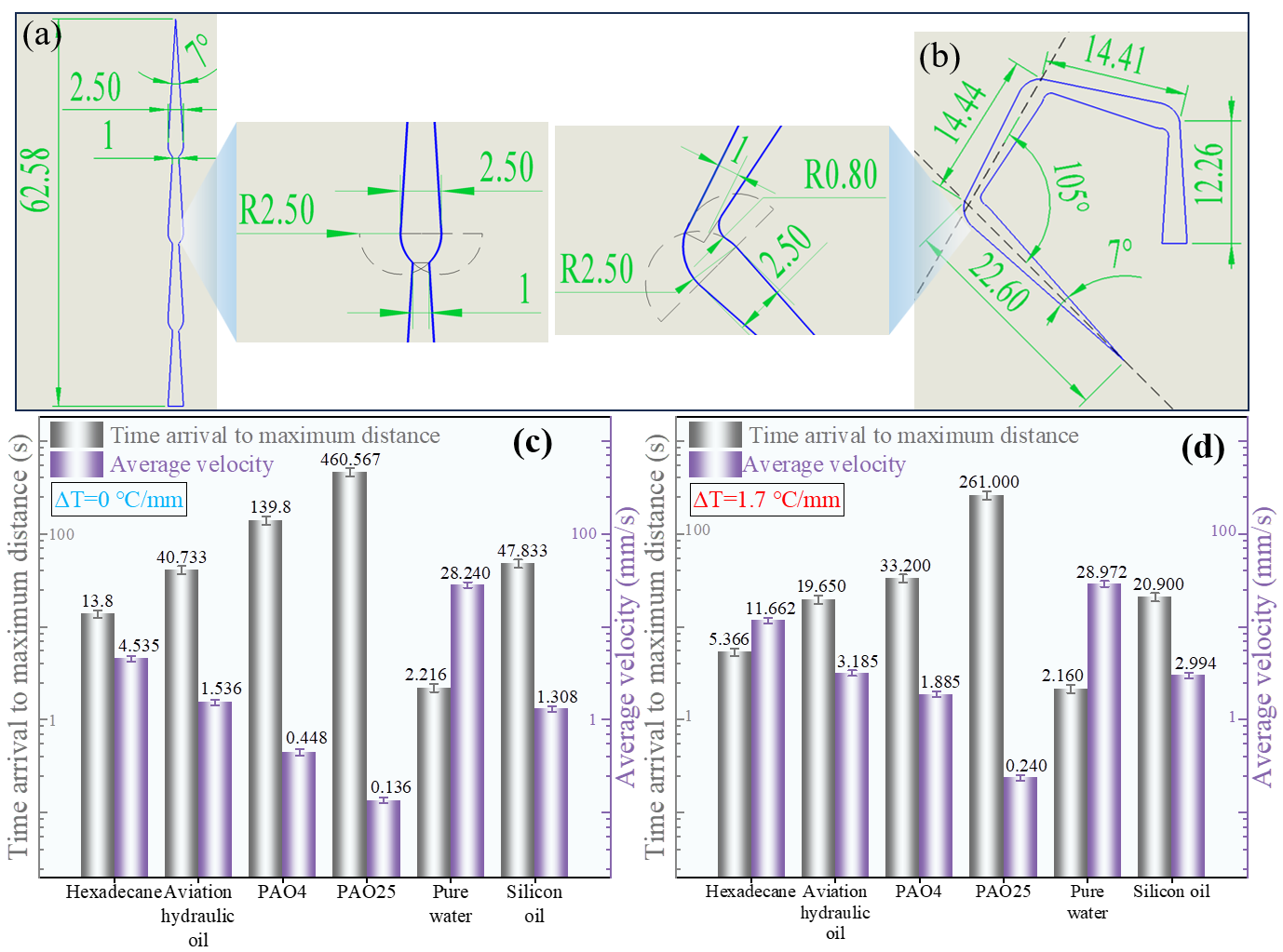


**Figure S1**. The dimensional design of the proposed structures in this study of (a) straight-wedged grooves pattern and (b) bent-edged grooves pattern. A visual and quantitative comparison of the transportation distance and velocity characteristics of these typical liquids under (c) ΔT= 0 ℃/mm and (d) ΔT= 1.7 ℃/mm.


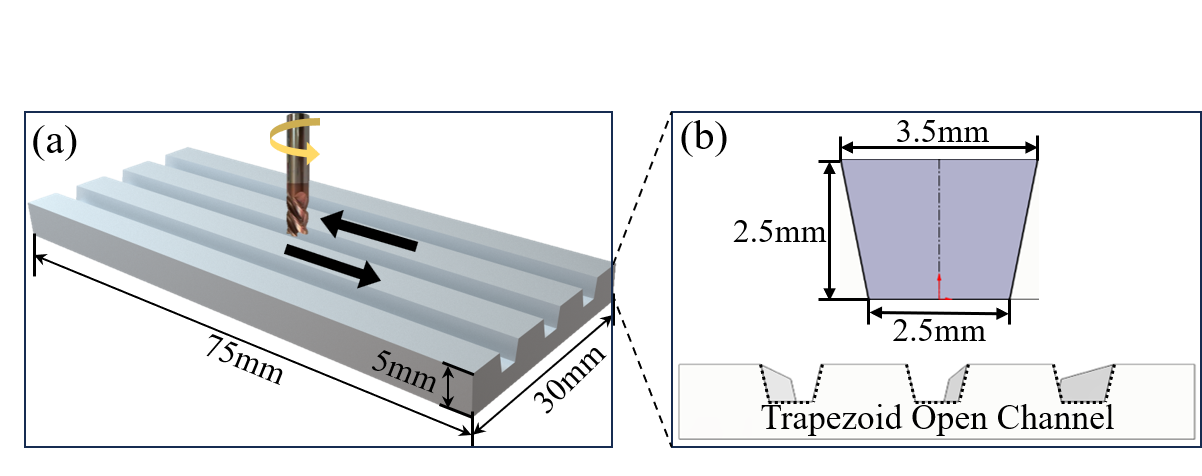


**Figure S2.** (a) Customized inverted trapezoid cutting tools for milling aluminum plate into trapezoid open channel, and the size of aluminum is 75×30×5mm. (b) The detailed size imagines of trapezoid open channel. The size of the up and bottom edges is 3.5mm and 2.5mm respectively, the height is 2.5mm, and the aspect ratio is 1. The purpose of designing a trapezoidal channel is that only the bottom edge of the channel will be laser etched after positioning. (It has been confirmed by experiment).


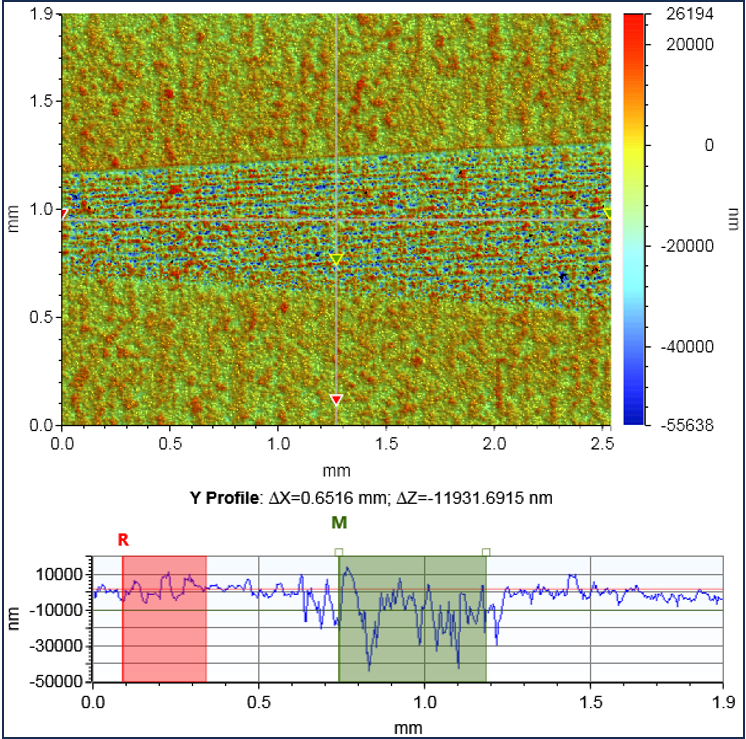


**Figure S3.** The surface topology of the optimized chamfer series wedge-shaped groove etched by one-time laser processing (The average depth is 11 μm captured by a three-dimension morphology instrument under 2.5x magnification, and the R and M regions in the figure both represent the numerical mean of the curve).


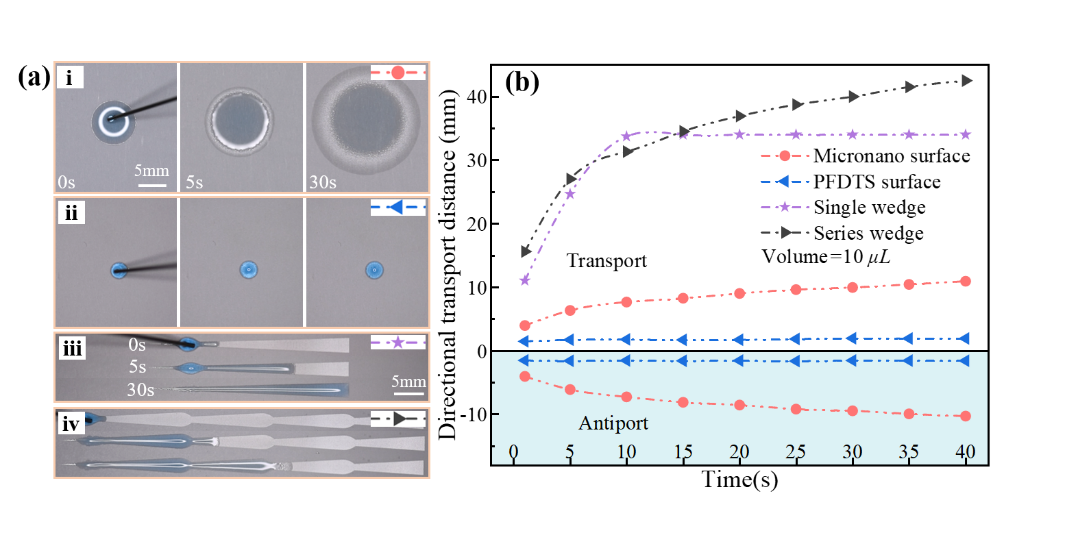


**Figure S4.** Self-transport phenomena of silicone oil (10μL) on free surfaces with different structures including micronano structures, PFDTS modification, single wedged groove, and wedged groove pattern: (a) transport phenomena, (b) detailed results.


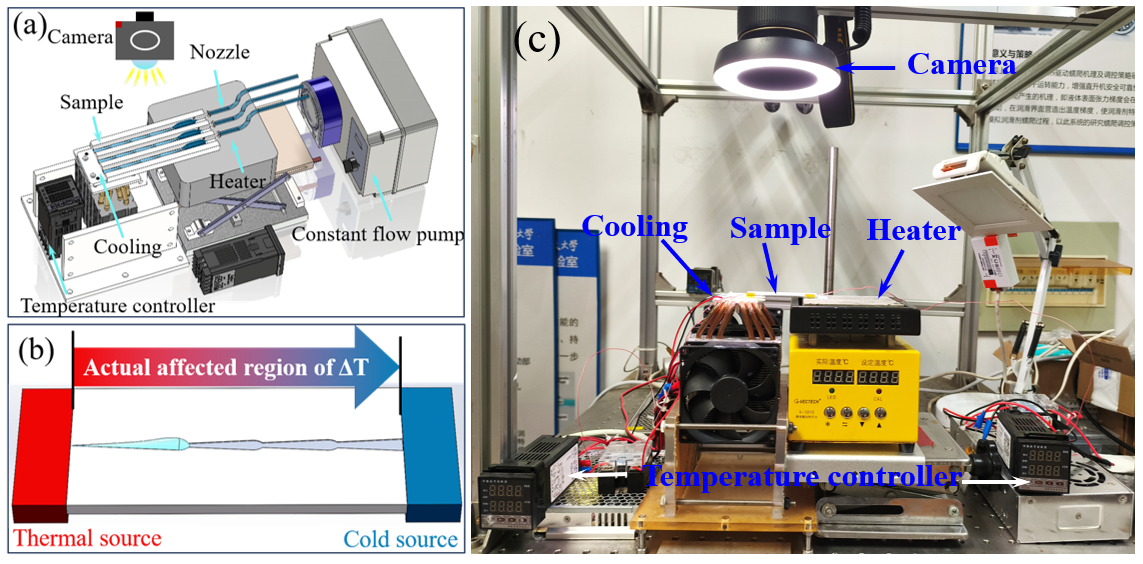


**Figure S5.** (a) Custom-designed experimental platform. Heating and cooling elements were assembled at the left and right sides under the specimen. The flow process was captured by the Camera. The heating one was a constant power heating platform (200 W), and the cooling one was a Peltier element (120 W). Regarding the heat absorption/release process of the aluminum substrate as a one-dimensional steady heat transfer process, ignoring the external heat loss and resistance to internal heat, the required time for changing the temperature of the aluminum substrate from *T_0_* to *T* can be determined as:$t=\frac{cm(T-T_{0})}{P}$ (*c* is the specific heat volume of aluminum, *c*≈0.9 J/g⋅℃, *m* is the mass, *m* ≈15 g, *T_0_* is the initial temperature, *T_0_*=25 ℃, *P* is the heating or cooling power). Thus, the required time for the heating process (*T*=150 ℃) is approximately 8.5s, and for the cooling process (*T*=0 ℃) is approximately 2.8s. The experimental setup is sufficient to heat or cool the aluminum substrate to the set temperature with the thermocouples and closed-loop control circuit.

The available length for experiments is 62.58 mm, via setting the temperature of the heating and cooling elements to 146 and 21 °C simultaneously, a thermal gradient of 2.0 °C/mm [(146-21) °C / 62.58 mm] could be generated along the length direction. The generated thermal gradient on the plate surface is confirmed by the thermal infrared imager (Ti32, Fluke, USA) and the J-type thermocouples (accuracy of ±0.75 °C).

**2. Movie Captions**

**Movie S1**

Performance of six different lubricants on surfaces with a wedged groove pattern (R = 2.5 mm) when ΔT = 0 °C/mm.

**Movie S2**

Performance of six different lubricants on surfaces with a wedged groove pattern (R = 2.5 mm) when ΔT = 1.7 °C/mm.

**Movie S3**

Synthesis and detection of reagents in a confined space: amino acid chromogenic reaction at room temperature and heated to 75 °C on surfaces with "double halberd" structures.

**Movie S4**

Transport processes on surfaces with capillary crura with orientation angles (*φ*) of 150°, 90°, and 30° when ΔT= 1.8 °C/mm.

**Movie S5**

Transport processes on four distinct surfaces including micronano structures, PFDTS modification, single wedged groove, and single wedged groove featuring capillary crura (*φ* = 30°).

**Movie S6**

Transport processes on two surfaces wedged groove pattern and wedged groove pattern with capillary crura (*φ* = 90°).

1. *Corresponding author: Qingwen Dai, daiqingwen@nuaa.edu.cn, ^1^C.D. and Q.D. contributed equally to this work. [↑](#footnote-ref-1)
